# Smartwatch gait coordination index: New measure for human gait utilizing smartwatch sensor

**DOI:** 10.1097/MD.0000000000033267

**Published:** 2023-03-24

**Authors:** Sumin Han, Rob Paul

**Affiliations:** a Korea International School, South Korea.

**Keywords:** gait, motion, phase coordination index, smartwatch

## Abstract

Human walking reflects the state of human health. Numerous medical studies have been conducted to analyze walking patterns and to diagnose disease progression. However, this process requires expensive equipment and considerable time and manpower. Smartwatches are equipped with gyro sensors to detect human movements and graph-walking patterns. To measure the abnormality in walking using this graph, we developed a smartwatch gait coordination index (SGCI) and examined its usefulness. The phase coordination index was applied to analyze arm movements. Based on previous studies, the phase coordination index formula was applied to graphs obtained from arm movements, showing that arm and leg movements during walking are correlated with each other. To prove this, a smartwatch was worn on the arms and legs of 8 healthy adults and the difference in arm movements was measured. The SGCI values with abnormal walking patterns were compared with the SGCI values obtained during normal walking. In the first experiment, the measured leg SGCI in normal walking averaged 9.002 ± 3.872 and the arm SGCI averaged 9.847 ± 6.115. The movements of both arms and legs showed stable sinusoidal waves. In fact, as a result of performing a paired *t* test of both exercise phases measured by the strike point using the maximum and minimum values, it was confirmed that the 2 exercises were not statistically different, as it yielded a *P* value of 0.469 (significance level *α* = 0.05). The arm SGCI measured after applying the 3 kg weight impairment on 1 leg was 22.167 ± 4.705. It was confirmed that the leg SGCI and 3 kg weight arm SGCI were statistically significant, as it yielded a *P* value of 0.001 (significance level *α* = 0.05). The SCGI can be automatically and continuously measured with the gyro sensor of the smartwatch and can be used as an indirect indicator of human walking conditions.

## 1. Introduction

Walking is one of the most fundamental and primary exercises performed by humans. Normal gait is often used as an indicator to determine abnormalities in walking patterns that are both directly and indirectly affected by health conditions.^[1–3]^ In other words, an abnormal gait is a direct signal of abnormalities in the human body. Typically, patients with Parkinson disease exhibit abnormal gait due to central neurological disorders. Detection of gait abnormalities plays a dominant role in predicting the progression of Parkinson disease and other neurological disorders. Recently, 3-dimensional analysis of these gait changes has been conducted in various ways, and researchers have been able to digitalize gait patterns.^[4–6]^ However, some limitations still exist. Gait analysis requires extremely expensive equipment, including multiple sensors and cameras, and a cumbersome process, and thus has constraints in measuring the long range of gait.^[7–10]^

**Figure 1.**
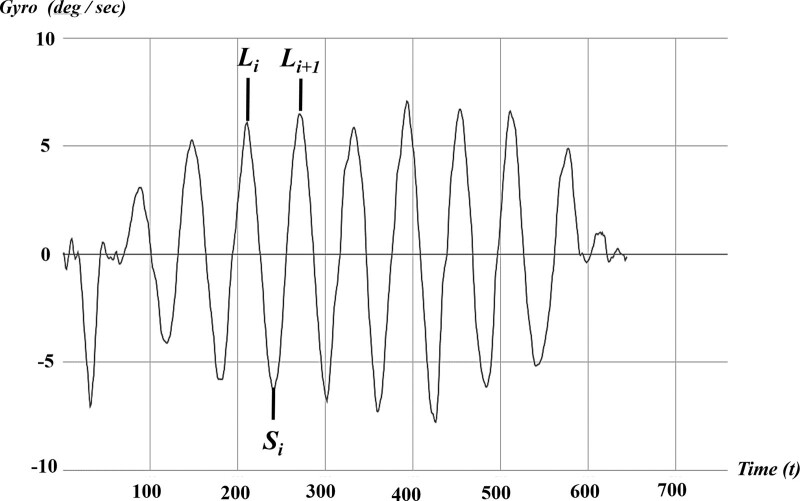
Arm movement pattern measured using a smartwatch. This graph depicts periodic and relatively constant movement of the sinusoidal wave motion. Li and Si: i-th time peak point at which the maximum (L) and minimum (22S) arm and leg motion.

Recently, however, smartwatches equipped with various heart rate sensors and physical sensors have become widely accessible to the public, improving the use of their highly developed health management functions. Furthermore, numerous studies related to the use of smartwatches in fitness have been conducted, such as measuring the time and rigor of exercises through the analysis of gyro sensors and 3-axis acceleration sensors. One function that this research paper will mainly focus on is the measurement of the pattern of arm-swing motion, which can be depicted by a continuous graph (Fig. 1). According to a recent study,^[11–13]^ arm movement and walking are interrelated, meaning that changes in arm-swinging motions can indicate changes in walking patterns. Referencing this study, this research paper aims to analyze the arm movement patterns using acceleration and gyro sensors of smartwatches to find out whether abnormalities exist in walking patterns. Furthermore, this study examined whether it is possible to replace the conventional analytical method with smartwatch sensor data, which quantifies the kinetic movement of the arm for detecting walking abnormalities.

## 2. Methods

This study is an observational study examining the smartwatch coordination index following the below process. This study was reviewed by the local Institutional Review Board (Yonsei Sarang Hospital IRB, Seoul, South Korea) and was approved for Institutional Review Board exemption because this study was a study based on data obtained from normal life.

### 2.1. Measuring smartwatch gait coordination index (SGCI)

The phase coordination index (PCI) proposed by Hausdorff^[14–16]^ is a measure used to examine variations in upper limb movement by comparing the swing times of one leg to the other. The PCI assumes that the walking movements of a healthy person are symmetric, meaning that the phase between each strike converges to 180°. Therefore, the complete cycle where one heel strike returns to that exact position in the next step is defined as 360°, and the phase of each strike is calculated using the ratio of the normalized step time and stride time:

(where t_(Li), t_(Si): time of the i-th time at which the heel of the foot meets the floor.)

PCI defines *φ* using the following formula, where 360° – 1 complete cycle – is multiplied by the ratio of the time of the short distance and the long distance between heel strikes in a cycle. A person with a normal walking pattern has a *φ* value closer to 180°.

In addition, the sum of the mean absolute difference (φ_ABS), which is the mean of the absolute differences of all *φ* from the standard degree of 180, and the coefficient of variation (φ_CV) are defined as a PCI, which is used as a measurement of walking motion.


$$\begin{array}{l} \varphi \_ABS=mean\;value\;of\;|\phi_{i}-180^{\circ }|s \end{array}$$

$$\begin{array}{l} \varphi \_CV=coefficient\;of\;variation\;of\;the\;mean\;of\varphi \end{array}$$

$$\begin{array}{l} PCI=\varphi \_ABS+\varphi \_CV \end{array}$$

The SGCI functions by applying the PCI equation to arm movement. When a person walks, both the legs and arms perform pendulum movements. According to previous studies, it has been proven that the pendulum movement of the leg is highly correlated with the pendulum movement of the arm. When the pendulum motion of the arm is measured using a smartwatch gyro sensor, a sinusoidal wave similar to that of walking can be obtained, where 1 gait cycle correlates with 1 cycle of the sinusoidal wave of the arm movement. Therefore, the gait phase *θ* is defined by replacing the strike point of 1 foot with the maximum point of the sinusoidal wave of the arm movement and the strike point of the opposite foot with the minimum point of the sinusoidal wave of the arm movement (Fig. 1).

SGCI, a measure of the extent of abnormality in arm wing motion, is defined as the sum of the mean absolute difference (θ_ABS) and coefficient of variation (θ_CV) in the gait phase θ.


$$\begin{array}{l} SGCI=\theta \_ABS+\theta \_CV \end{array}$$

### 2.2. Experiments to measure arm and leg motion using smartwatch

**Figure 2.**
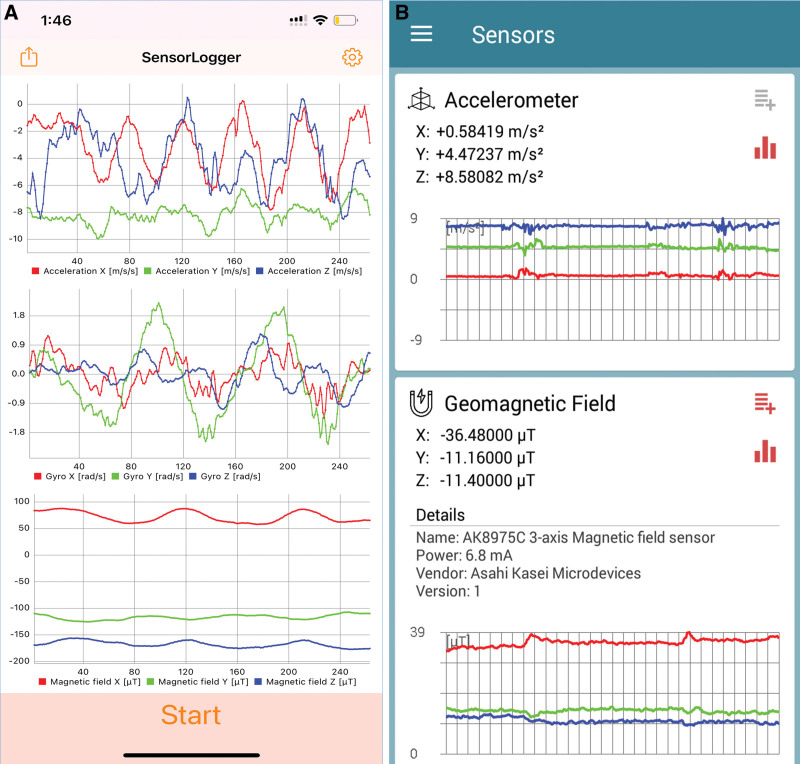
The example real sinusoidal graph of actual measured arm and leg movement using (A) Apple watch and (B) Galaxy watch. (A) This is the motion graph of the Apple watch extracted by the Sensor logger program. The upper graph is a graph showing the movement of the watch divided by the x, y, and z axes, and the movement of arms and legs was measured using an X-axis graph showing vertical movement (red color graph). (B) This is a motion graph of the Galaxy watch extracted by the Sensors Toolbox program. The accelerometer graph at the top is a graph showing the movement of an accelerometer mounted on a clock divided by x, y, and z axes, and the movement of arms and legs was measured using a Gyro Z-axis graph showing vertical movement.

One Apple watch and 1 Galaxy watch were used for this experiment. The Apple watch was applied around the ankle and the Galaxy watch around the wrist during gait and the gait analysis from both watches was extracted. The graph and the data from the Apple watch were extracted by connecting the Apple developer directly to the laptop using the Sensor Logger program, while the data from the Galaxy watch was extracted with the Sensors Toolbox program which is a basic application. The frequency of the graph was 20 Hz and 120 Hz each. The X-axis sensor data of the apple watch was used while the Gyro-z sensor data from the Galaxy watch (Fig. 2).

The initiation and the termination of a movement do not represent the exact gait so the first 5 steps were excluded, and the maximum amplitude was hypothesized at 1 while the section below the amplitude of 0.76 was also excluded for data analysis. To earn the strike point (t(S_i), t_(L_i)) for the calculation of the phase of each strike, the PCI method of Hausdorff uses the moment when the feet contact the ground whereas the SGCI method proposed in this study is not based on videography so the apex of the arm movement cannot be extracted exactly. To compromise the limitation, the SGCI method uses the extreme point of the arm movement graph extracted from the smartwatch. Most movement graph has 1 maximum and minimum point each but some have several (mostly 2). This study adapts the maximum and the minimum of the existing extreme points for the simplicity of analysis.

The maximum and minimum values were obtained using a self-produced coding program from the graph obtained by the gait experiment, and SGCI was calculated using the maximum and maximum values of >20 steps.

### 2.3. Human data evaluation

During the research, we attempted to prove the scientific basis of SGCI by conducting 2 experiments: one, aiming to prove that the exercise of arms and legs are coupled when walking, and another, aiming to prove whether the SGCI measured with the smartwatch can detect gait abnormality.

Gait analysis was conducted on 8 healthy men aged between 22 and 27. First, the test subjects wore a smartwatch on their wrist and ankle while walking normally, and the movements of their legs and arms were analyzed using the data recorded by the acceleration and gyro sensors of the smartwatch. The subjects walked over 8 steps on a 10-meter passage, and the walking data of the entire section were recorded. Walking data with an amplitude within 10% of the maximum amplitude, which continued for at least 3 cycles were used for the actual analysis.

In addition, to represent abnormal walking, elements that caused walking abnormalities were added to subjects with normal walking patterns. More specifically, these subjects imitated people with walking abnormalities by carrying a 3 kg sandbag on 1 ankle. Subsequently, we analyzed and compared the patterns of arm movements during walking and normal walking.

First, we analyzed the correlation between leg SGCI (when subjects wore smartwatches on their ankle) and arm SGCI (when subjects wore smartwatches on their wrist) during a normal walking state. Second, we obtained and analyzed the 3 kg weight arm SGCI values during an abnormal walking state.

All data were statistically evaluated by performing the paired *t* test of both exercise phases measured by the strike point using the maximum and minimum values.

## 3. Results

At first, our data set was evaluated if this showed a normal distribution. Using Kolmogorov–Smirnov test and Shapiro–Wilk test, our data showed normal distribution. The *P* values of leg SGCI, arm SGCI, and 3 kg weight arm SGCI were 0.26, 0.121, and 0.142 and 0.096, 0.033, and 0.284 each. According to these results, we performed a paired *t* test for analysis.

**Figure 3.**
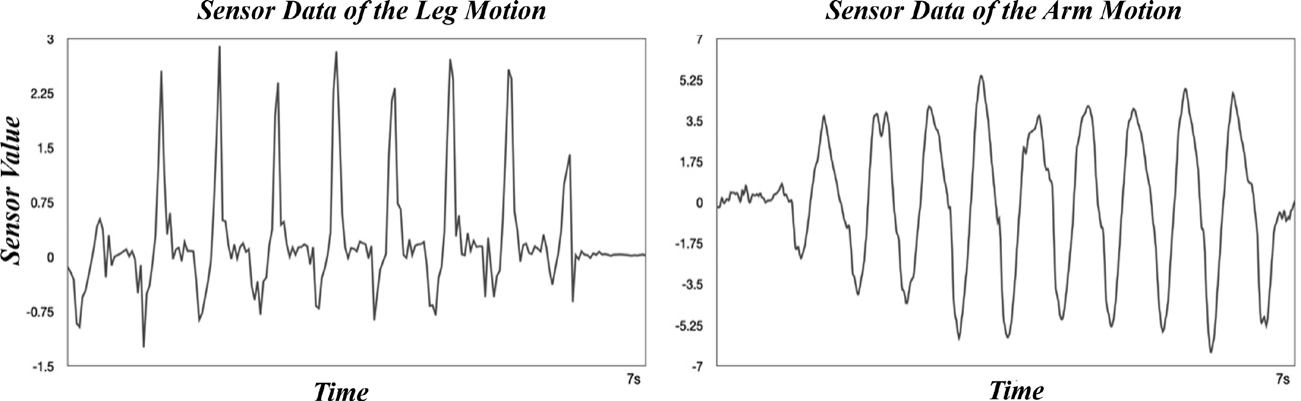
Gyro-sensor graph of leg movement (left) and gyro-sensor graph of leg movement arm (right) which show similar sinusoidal wave patterns during normal gait.

In the first experiment, the measured leg SGCI values in normal walking averaged 9.002 ± 3.872. The arm SGCI averaged 9.847 ± 6.115 (Table 1). The gyro sensor graph measuring the movement of the subjects’ arms and legs during a normal walking state is as follows. Both movements exhibited stable sinusoidal waves (Fig. 3). In fact, as a result of performing the paired *t* test of both exercise phases measured by the strike point using the maximum and minimum values, it was confirmed that the 2 exercises were not statistically different, as it yielded a *P* value of 0.079 under the significance level *α* = 0.05.

**Table 1:** Results of 8 samples SGCI of human gait and arm swing.

Subject	Leg SGCI	Arm SGCI	3 kg SGCI
1	14.896	20.168	22.370
2	6.604	7.746	13.836
3	4.535	5.220	25.547
4	6.630	4.435	24.282
5	14.397	18.487	16.427
6	7.095	9.947	24.741
7	7.174	5.537	22.155
8	10.681	7.231	27.651
Average	9.002	9.846	22.126
SD	3.872	6.115	4.705

*P* value: *P* = .484, between leg SGCI and Arm SGCI.

*P* value: *P* = .001, between arm SGCI and 3 kg arm SGCI.

Leg SCGI: SGCI of 1 leg during normal walking.

Arm SGCI: SCGI of 1 arm during normal walking.

3 kg SGCI: SGCI of arm during walking with 3 kg weight on 1 leg.

SD = standard deviation, SGCI = smartwatch gait coordination index.

**Figure 4.**
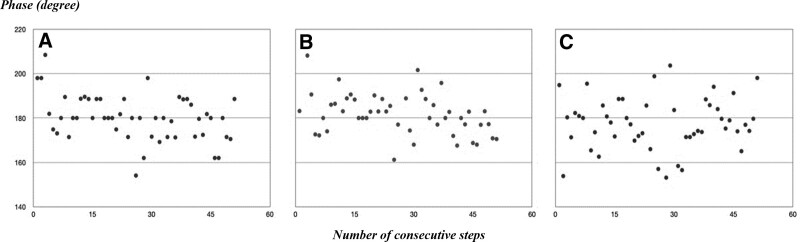
The scatter plots of phase (degree) for consecutive steps. (A) Movement of legs during normal walking. (B) Movement of arms during normal walking. (C) Difference between movement of arms and movements of legs during normal walking.

The 3 kg weight arm SGCI measured after applying the 3 kg weight impairment on 1 leg was 22.167 ± 4.705 (Table 1). The asymmetric appearance of the SGCI graph, where the points are concentrated on the left, indicates that the gait phase is biased towards 1 side (Fig. 4). Furthermore, the value of the SGCI with gait disorder conditions was analyzed using the paired *t* test and it showed statistically different from that measured without gait disorder conditions. It was confirmed that the leg SGCI and 3 kg weight arm SGCI were statistically significant, as they yielded a *P* value of 0.001 under the significance level *α* = 0.05 (Table 1).

## 4. Discussion

One of the simplest methods for measuring gait patterns is the method proposed by M. Jeffrey M. Hausdorff et al,^[14–16]^ which quantifies the bilateral coordination of gait using the difference between the time when the foot touches the ground and when the foot is off the ground. To summarize, from the moment when the foot leaves the ground to the moment when it touches the ground again is defined as 2π rad, and the time ratio of the exact moment a person’s foot leaves the ground and when it touches the ground again is defined as φ. Using this method, the sum of the absolute difference and coefficient of variation for the standard 180° of 1 leg is defined as the PCI, which is used as a measure of abnormality in a walking motion. Currently, this measurement is applied in numerous fields to evaluate walking pattern abnormalities, such as Parkinson disease.^[13–15]^ However, the initially proposed PCI-calculated gait measurement method is cumbersome and costly. Therefore, a simple, approachable, and inexpensive method for walking analysis is required. As an alternative, this research paper proposes measuring walking phases using walking impairment measurement, SGCI, in gyro sensors of smartwatches that are easily accessible to the public.

The SGCI proposed in this study is an automatically measured method using smartwatch gyro sensors without the need for measurements that must be carried out by a human himself. The time used in the measurement was not when the heel touched the ground but when the wrist reached its highest point when walking. When a person walks using 2 legs, the arm exercises simultaneously according to the movement of the leg, as it performs a swing motion. At this point, the wrist performs a pendulum movement with the longest radius and reaches its peak at approximately the same time as the heel tread, and again, the pendulum swings at approximately the same period as the foot exercise cycle. The results of this study show that when measuring the gyro sensor of the smartwatch during walking exercise, the wrist movement was precisely reflected in the sensor, and the time when the highest point of the wrist and the maximum point of the sinusoidal wave were almost identical. Therefore, the SGCI can indirectly measure gait motion in the same manner as Hausdorff proposed coordination by setting the peak of the wave as the time measurement in the smartwatch gyro sensor.

Many medical studies have verified that the movement of the feet and the movement of the arms during bipedalism have great connectivity with each other.^[11–13]^ When walking, the movement of the upper body, especially the movement of the arm, is directly influenced by the rotational force caused by the movement of the lower body, meaning that the upper body rotates simultaneously while coordinating the movements of the legs. In particular, the rotational movement of the upper body proceeds in the opposite direction to that of the lower body, which plays a major role in maintaining the balance of the body during walking. In a study using an actual pendulum model, it was shown that there was a high correlation between arm movement and leg movement when walking.

As a result of the experiment conducted in this study, SGCI was able to measure gait disability relatively similar to PCI, which is expected to be highly utilized in today’s world. It is possible to indirectly detect changes in walking by wearing a smartwatch in daily life without having to visit a specific facility to measure gait disorders. Abnormal changes in SGCI may be able to selectively warn that kinematic dysfunction is present in the gait. In addition, a program can be developed by applying this concept, and by installing it in a healthcare application, it will be possible to easily measure the presence and predict the progress of gait impairment in daily life.

The limitation of this study was small subjective numbers due to the pilot study but will be evaluated with large numbers.

## 5. Conclusion

In conclusion, the SCGI can be automatically and continuously measured using the gyro sensor of the smartwatch, and the measured SCGI can be used as an indirect indicator of walking conditions.

## Author contributions

**Conceptualization:** Sumin Han.

Data curation: Sumin Han.

Formal analysis: Sumin Han.

Investigation: Rob Paul.

Methodology: Rob Paul.

Validation: Rob Paul.

Writing – original draft: Sumin Han.

Writing – review & editing: Sumin Han.
